# Outcomes With a Mobile Digital Health Platform for Patients Undergoing Spine Surgery: Retrospective Analysis

**DOI:** 10.2196/38690

**Published:** 2022-10-26

**Authors:** Vishal Venkatraman, Elayna P Kirsch, Emily Luo, Sameer Kunte, Madison Ponder, Ziad F Gellad, Beiyu Liu, Hui-Jie Lee, Sin-Ho Jung, Michael M Haglund, Shivanand P Lad

**Affiliations:** 1 Department of Neurosurgery Duke University Medical Center Durham, NC United States; 2 Higgs Boson Health Durham, NC United States; 3 Department of Biostatistics & Bioinformatics Duke University School of Medicine Durham, NC United States

**Keywords:** digital health, spine surgery, surgical outcomes, mobile health, mobile application, surgery, postoperative, mobile health, mobile app, mHealth, recovery

## Abstract

**Background:**

Digital health solutions have been shown to enhance outcomes for individuals with chronic medical illnesses, but few have been validated for surgical patients. The digital health platform ManageMySurgery (MMS) has been validated for spine surgery as a feasible method for patients along their surgical journey through in-app education and completion of patient-reported outcomes surveys.

**Objective:**

The aim of this study is to determine the rates of 90-day emergency room (ER) visits, readmissions, and complications in patients undergoing spine surgery using MMS compared to patients using traditional perioperative care alone.

**Methods:**

Patients undergoing spine surgery at a US-based academic hospital were invited to use MMS perioperatively between December 2017 and September 2021. All patients received standard perioperative care and were classified as MMS users if they logged into the app. Demographic information and 90-day outcomes were acquired via electronic health record review. The odds ratios of having 90-day ER visits, readmissions, mild complications, and severe complications between the MMS and non-MMS groups were estimated using logistic regression models.

**Results:**

A total of 1015 patients were invited, with 679 using MMS. MMS users and nonusers had similar demographics: the average ages were 57.9 (SD 12.5) years and 61.5 (SD 12.7) years, 54.1% (367/679) and 47.3% (159/336) were male, and 90.1% (612/679) and 88.7% (298/336) had commercial or Medicare insurance, respectively. Cervical fusions (559/1015, 55.07%) and single-approach lumbar fusions (231/1015, 22.76%) were the most common procedures for all patients. MMS users had a lower 90-day readmission rate (55/679, 8.1%) than did nonusers (30/336, 8.9%). Mild complications (MMS: 56/679, 8.3%; non-MMS: 32/336, 9.5%) and severe complications (MMS: 66/679, 9.7%; non-MMS: 43/336, 12.8%) were also lower in MMS users. MMS users had a lower 90-day ER visit rate (MMS: 62/679, 9.1%; non-MMS: 45/336, 13.4%). After adjustments were made for age and sex, the odds of having 90-day ER visits for MMS users were 32% lower than those for nonusers, but this difference was not statistically significant (odds ratio 0.68, 95% CI 0.45-1.02; *P*=.06).

**Conclusions:**

This is one of the first studies to show differences in acute outcomes for people undergoing spine surgery who use a digital health app. This study found a correlation between MMS use and fewer postsurgical ER visits in a large group of spine surgery patients. A planned randomized controlled trial will provide additional evidence of whether this digital health tool can be used as an intervention to improve patient outcomes.

## Introduction

Approximately 35,000 cervical spine procedures and 200,000 lumbar spine surgeries are performed in the United States each year [1,2]. This high surgical volume is also associated with significant resource use in the health care system, with expenses averaging around US $20,000 for cervical cases and US $50,000 for lumbar cases.

Recent changes to the American Medicare and Medicaid insurance systems have rewarded hospitals for reducing 90-day postoperative emergency room (ER) visits and penalized them for unnecessary ER visits [3]. The rates of 90-day postoperative ER visits at 2 major medical centers were 9.4% and 13%, respectively, with postoperative discomfort being the most common reason [3,4]. According to one of these centers’ economic analyses, the average postoperative spine ER visit costs around US $2000, whereas the average readmission costs US $7400. [3] Given the significant economic burden, improved patient follow-up and education about medical emergencies may help minimize health care resource utilization by reducing postoperative ER visits following spine surgery.

Furthermore, spine procedures are linked with high clinical morbidity. Postoperative morbidity rates for cervical fusion and cervical arthroplasty have been reported to be as high as 19%-20% [5,6], with common complications including wound infections, dysphagia, hematoma, and urine retention [5-7]. Lumbar procedures include laminectomies and discectomies, as well as more traditional posterior fusion techniques (transforaminal and posterior lumbar interbody fusions) and less invasive anterior and lateral interbody fusions [8-10]. Complication rates for lumbar techniques vary greatly, with some studies showing rates of 14% for anterior lumbar fusion [11], 30%-40% for severe lateral lumbar fusion [12], and 8%-17% for transforaminal and posterior lumbar fusion [9,11]. Many patients may be fearful about spine surgery due to the variety of surgical methods, the complexity of the anatomy, and the variety of possible postoperative outcomes. With the increasing number of outpatient procedures, there is an increasing unmet need to assist patients in navigating these complex spine therapies and achieving the best potential outcomes.

Prior research on postoperative ER visits has discovered that early postoperative phone calls and telehealth visits from clinical personnel can help minimize ER visits [13,14], implying that early patient engagement and communication can help alleviate the health care system’s burden. Although telehealth visits and phone calls may alleviate some of the strain on health care resources, they are time-consuming and difficult to scale.

Mobile apps are being aggressively deployed as platforms for connecting care professionals and patients and for providing information outside of the hospital setting in today's increasingly digital environment where smartphone use is common. A review of mobile health solutions reveals an abundance of new apps for patient education, clinical diagnostics, treatment adherence, and behavioral change [15]. Mobile health apps have been shown to reduce patient visits and hospitalizations and to facilitate self-care in patients with chronic conditions such as diabetes and cardiovascular disease [16,17]. Certain mobile apps have been designed to serve as acute perioperative care tools for communicating pre- and postoperative instructions and concerns. Apps designed for abdominal and orthopedic procedures reduce follow-up visits [18-20].

Previously, our team proved the feasibility of ManageMySurgery (MMS), a perioperative mobile app, in educating patients across various interventional and surgical paths and in gathering patient-reported outcomes for spine and breast procedures [21,22]. MMS enables patients to access instructional content tailored to their procedure, receive notifications and reminders along standardized care pathways and from their provider teams, and complete pre- and postoperative questionnaires to inform and monitor their clinical team.

Although digital health is rapidly expanding, little research has been done on its quantifiable impact on patient outcomes. We anticipate that using a complete digital health platform specialized for spine surgery (MMS) can help patients undergoing elective spine surgery avoid emergency department visits, postoperative hospital readmissions, and postoperative problems. We specifically hypothesize that compared to patients using traditional perioperative care, those using MMS will have fewer ER visits, fewer hospital readmissions, and fewer postoperative complications in the 90 days after surgery. This would directly impact the health care cost placed on patients having these procedures and could also reduce the systemwide burden of excess health care resource utilization.

## Methods

### Ethics Approval

Ethics approval was obtained prior to beginning the study from the Duke University Institutional Review Board (protocol #Pro00074329).

### Description of the MMS App

MMS is a cloud-based, HIPAA (Health Insurance Portability & Accountability Act)-compliant solution that provides a platform for patients and caregivers undergoing interventional or surgical procedures. It allows patients to prepare for procedures through in-app educational content specific to their surgery, access to frequently asked questions (FAQs), and communication with their surgical team. The MMS-Spine module supports the most commonly performed spine surgeries, with submodules available for lumbar laminectomy or discectomy, lumbar fusion, and anterior cervical discectomy and fusion. The app is available on mobile operating systems including Android (Google Inc) and iOS (Apple Inc), as well as through a web app to reach the widest patient population possible.

MMS was designed by an interdisciplinary team, and the spine module described in this study (MMS-Spine) uses evidence-based guidelines from national societies, including the North American Spine Society, American Association of Neurological Surgeons, and the American Association of Orthopedic Surgeons. The app phrases questions, responses, and other content at a sixth grade reading level. Literacy evaluation was performed by the Duke Patient Education Governance Council.

A nurse navigator creates an account for patients who are invited to use MMS-Spine, enters patient demographic information into the app, and assigns them to a submodule based on surgery type: Anterior Cervical Discectomy & Fusion (ACDF), Spinal Fusion, or Lumbar Discectomy. These function as care pathways that contain different sets of educational materials and tasks specific to the surgery type. As an example, some FAQs within the ACDF submodule are “What are the risks of ACDF?” and “What is the process for getting an ACDF?”. In the Spinal Fusion module questions include “How will a spinal fusion affect my flexibility or ability to move?” and “What are the risks of spinal fusion?” [21]. Patients can also view postoperative information, such as serious symptoms to watch for during recovery and restrictions on activity, eating, and drinking after surgery [4].

MMS also collects patient-reported outcomes via in-app surveys, specifically the commonly used and well-validated PROMIS-29 (Patient-Reported Outcomes Measurement Information System 29), Oswestry Back Disability Index, and Neck Disability Index [23-26]. Baseline surveys are collected 2 to 4 weeks prior to surgery, and postoperative surveys are automatically available to patients after discharge, with automatic reminders delivered to their smartphones to complete these surveys. Results from these surveys are analyzed and presented separately. Patients are also prompted with reminders to complete other tasks, such as checking into appointments and completing preoperative instructions.

Providers can view results of the MMS app as well as the responses and trends from the patient surveys. Furthermore, patients and providers can communicate within the application through a HIPAA-compliant messaging system. Multimedia Appendix 1 and Multimedia Appendix 2 contain screenshots of the app, including the patient and provider interfaces.

### Participants and Setting

This project was conducted as a retrospective cohort study. Patients were eligible for the study if they were scheduled to undergo an elective spine surgery at Duke University Health System between December 2017 and September 2021, English was their primary language, they were at least 18 years old, they had a device capable of running MMS (iOS, Android, or desktop computer), they could consent, their surgeon had invited them to join MMS during a preoperative appointment, and if the patient had at least 90 days of follow-up after their surgical procedure. Patients without phones who wanted a family member or friend to proxy using the in-app caregiver function were also invited to use MMS and were included in the study. Patients were excluded if 90 days had not passed since their surgery date and if they had surgery at more than 6 spinal levels, as these surgeries were typically for scoliosis or other deformity procedures that were not supported by the MMS app at the time.

If the patients accepted their invitation by logging into the app, they were assigned to the MMS user cohort. Nonusers were considered to be those patients or their designated caregivers who had never logged into MMS.

Users of MMS downloaded and logged into the app 2 to 4 weeks before the elective spine surgery, received structured preoperative information, and completed baseline surveys. Patients could complete 6-week, 3-month, 6-month, and 12-month postoperative surveys after surgery. Patients received push notifications via the app to complete these surveys, check into appointments, and complete other tasks assigned to them by their provider team. Consent was obtained electronically and at the time of enrollment. Consent included permission to use MMS app and electronic health record outcomes and demographic data for research purposes. Each patient completed a brief, standardized, self-guided walk-through orientation within the app, which included instructions on how to access educational materials and complete tasks (such as completing surveys or checking into appointments). This procedure was also followed by proxies who used the caregiver function.

### Data Collection

MMS collected and securely stored data gathered throughout the patient's engagement with the app using Amazon Web Services. The MMS database was used to collect app usage data. The app collects data such as the number of account sign-ins, task or survey completion, the addition of proxy caregiver(s), the device used to access MMS, and the number of FAQs viewed.

A chart review from the electronic health record was used to collect patient demographics (age at time of procedure, sex, insurance status), surgical details (specific procedure, number of spinal levels), and clinical outcomes within 90 days of surgery. The specific 90-day clinical outcomes collected in this study included postoperative unplanned readmissions to any hospital, excluding other preplanned admissions such as postoperative rehabilitation, colonoscopy, other elective surgeries, postoperative ER visits at any hospital, reasons for these postoperative ER visits, and postoperative complications.

Complications were ranked in severity using the Clavien-Dindo scale, which has been validated for spine surgery [27,28]. The Clavien-Dindo scale ranks postoperative complications from 1 to 5, with 1 indicating mild or no treatment needed, 2 indicating complications requiring pharmacologic treatments or blood transfusions, 3 indicating procedural treatment (surgery, interventional radiology, endoscopy), 4 indicating intensive care unit–level treatment or organ failure, and 5 indicating patient death [29]. For this study, the Clavien-Dindo score was further used to classify patients into the categories of mild complication (Clavien-Dindo score 1-2) or severe complication requiring intervention (Clavien-Dindo score 2-5)

### Statistical Analysis

Descriptive statistics are used to summarize patient demographics, surgical characteristics, MMS usage, and reasons for ER visits. Means, SDs, medians, first and third quartiles, and minimum and maximum values are reported for continuous variables. The number and percentage of nonmissing values for categorical variables are reported. The 90-day ER visit rate, 90-day readmission rate, 90-day mild complication rate, and 90-day severe complication rate, as well as their 95% CIs using a binomial distribution, are reported as primary outcomes. Along with adjustments for age and sex, multinomial logistic regression models were used to estimate the odds ratio of having a 90-day ER visit, 90-day readmission, 90-day mild complication, and 90-day severe complication in the MMS group versus the non-MMS group. All statistical tests were 2-sided, and test significance was determined at α=.05 without accounting for multiple testing. SAS version 9.4 (SAS Institute Inc) was used for statistical analysis.

## Results

### Patient Characteristics and MMS App Usage

A total of 1160 patients undergoing elective spine surgery at Duke University Medical Center were invited to use MMS between December 2017 and September 2021. After inclusion and exclusion criteria of age, minimum follow-up time, and number of surgical levels were applied, 1015 patients were included in the final study cohort. Of this cohort, 679 patients or their caregivers (66.90%) logged into MMS at least once and were considered MMS users, while 336 patients (33.10%) did not use MMS.

Table 1 shows the demographics of the patients. Patients in both groups were of similar age (non-MMS: mean 61.5 years, SD 12.7 years; MMS: mean 57.9 years, SD 12.5 years) and had equal proportions of uninsured patients (MMS: 12/679, 1.8%; non-MMS: 6/336, 1.8%). The MMS group had more males (MMS: 367/669 54.1%; non-MMS: 159/336, 47.3%). Patients in both groups most commonly underwent cervical fusion operations (559/1015, 55.07%) or single-approach lumbar fusions (231/1015, 22.75%). Additionally, 360 patients (360/1015, 35.46%) underwent single-level operations, and 341 patients (341/1015. 33.60%) underwent 2-level operations.

MMS usage is summarized in Table 2. The MMS app was used by 679 patients, with 397 (58.5%) using an iOS device and 253 (37.2%) using an Android device. The ACDF module was used by 387 (57%), the discectomy module by 65 (9.6%), and the spinal fusion module by 227 (33.4%). Patients and their caregivers logged onto MMS an average of 3.4 (SD 4) times; however, patients could access the app multiple times per login until they are logged out; thus, actual usage was likely higher. Moreover, 236 (34.8%) patients gave access to proxy caregivers, of whom 188 (79.7%) logged in to use the app, and 50.2% of patients viewed at least 1 FAQ.

**Table 1:** Demographic and clinical characteristics of patients.

Patient characteristics		Non-MMS^a^ (n=336)	MMS (n=679)	Total (N=1015)
Age at surgery (years), mean (SD)		61.5 (12.7)	57.9 (12.5)	59.1 (12.7)
Age at surgery (years), median (Q1, Q3^b^)		62 (54, 71)	58 (49, 68)	60 (50, 69)
Age at surgery (years), range		19-88	22-88	19-88
**Patient sex, n (%)**				
	Male	159 (47.3)	367 (54.1)	526 (51.8)
	Female	177 (52.7)	312 (45.9)	489 (48.2)
**Payor group, n (%)**				
	Commercial	129 (38.4)	336 (49.5)	465 (45.8)
	Medicare	169 (50.3)	276 (40.6)	445 (43.8)
	VA^c^/military/government employee/Medicaid	32 (9.5)	55 (8.1)	87 (8.6)
	None	6 (1.8)	12 (1.8)	18 (1.8)
**Procedure, n (%)**				
	360 lumbar fusion^d^	32 (9.5)	70 (10.3)	102 (10.0)
	Cervical fusion^e^	166 (49.4)	393 (57.9)	559 (55.1)
	Laminectomy/discectomy	40 (11.9)	64 (9.4)	104 (10.2)
	Lumbar fusion^f^	90 (26.8)	141 (20.8)	231 (22.8)
	Other^g^	8 (2.4)	11 (1.6)	19 (1.9)
**Surgery levels, n (%)**				
	1	108 (32.1)	252 (37.1)	360 (35.5)
	2	121 (36.0)	220 (32.4)	341 (33.6)
	3	67 (19.9)	130 (19.1)	197 (19.4)
	4	33 (9.8)	67 (9.9)	100 (9.9)
	5	5 (1.5)	7 (1.0)	12 (1.2)
	6	2 (0.6)	3 (0.4)	5 (0.5)
Surgery level, median (Q1, Q3)		2 (1, 3)	2 (1, 3)	2 (1, 3)

^a^MMS: ManageMySurgery app.

^b^Q1, Q3: first and third quartiles.

^c^VA: Veterans Affairs.

^d^Anterior approach to lumbar fusion + posterior approach to lumbar fusion.

^e^Includes both anterior and posterior cervical fusions.

^f^Includes singular approach to lumbar fusion, anterior or posterior.

^g^Includes cervical arthroplasty, thoracic fusion, sacroiliac fusion.

**Table 2:** MMS app usage results (n=679).

Characteristic			Value
**Patients and caregivers’ total sign-in counts, n (%)**			
	1-3	470 (69.2)	
	4-6	137 (20.2)	
	7+	72 (10.6)	
Patients and caregivers’ total sign-in counts, mean (SD)			3.4 (4)
Patients and caregivers’ total sign-in counts, median (Q1, Q3)			2 (1, 4)
Caregiver added, n (%)			236 (34.8)
Added caregivers that logged in (out of 236), n (%)			188 (79.7)
**Device used, n (%)**			
	iOS	397 (58.5)	
	Android	253 (37.2)	
	Web app/other	29 (4.3)	
**MMS^a^ submodule used, n (%)**			
	ACDF^b^	387 (57.0)	
	Lumbar discectomy	65 (9.6)	
	Spinal fusion	227 (33.4)	
**Number of FAQs^c^ viewed, n (%)**			
	0	338 (49.8)	
	1-10	97 (14.3)	
	11-20	75 (11.0)	
	21-40	104 (15.3)	
	41+	65 (9.6)	
Number of FAQs viewed, mean (SD)			12.1 (18.2)
Number of FAQs viewed, median (Q1, Q3^d^)			1 (0, 20)
Number of FAQs viewed, range			0-93

^a^MMS: ManageMySurgery app.

^b^ACDF: Anterior Cervical Discectomy & Fusion.

^c^FAQs: frequently asked questions.

^d^Q1, Q3: first and third quartiles.

### Ninety-Day Clinical Outcomes

Table 3 displays the 90-day ER visit rates, readmission rates, and postoperative complication rates. Of the 336 MMS nonusers, 30 (8.9%) had a readmission, 45 (13.4%) had an ER visit, 32 (9.5%) had a mild complication, and 43 (12.8%) had a severe complication within 90 days of their initial operation. Among the 679 patients who used MMS, there were 55 (8.1%) readmissions, 62 (9.1%) ER visits, 56 (8.3%) mild complications, and 66 (9.7%) severe complications.

As shown in Table 3, MMS patients were significantly less likely than non-MMS patients to have a 90-day ER visit with a univariable odds ratio of 0.65 (95% CI 0.43-0.98; *P*=.04). The odds ratio for an MMS patient having a 90-day readmission compared to a non-MMS patient having a 90-day readmission was 0.90 (95% CI 0.56-1.43; *P*=.65). The odds ratio for an MMS patient having a 90-day severe complication compared to a non-MMS patient was 0.72 (95% CI 0.48-1.09; *P*=.12), while the odds ratio for a mild complication was 0.82 (95% CI 0.52-1.3; *P*=.40).

When adjusted for age and sex, the odds ratio for an MMS patient having a 90-day readmission compared to a non-MMS user was 0.97 (95% CI 0.6-1.55; *P*=.88), the odds ratio for a 90-day severe complication was 0.78 (95% CI 0.52-1.19; *P*=.25), the odds ratio for a 90-day mild complication was 0.95 (95% CI 0.59-1.51; *P*=0.82), and the odds ratio for a 90-day ER visit was 0.68 (95% CI 0.45-1.02; *P*=.06).

Among the 107 patients in both groups who visited the ER, the most common reasons for an ER visit included syncope or falls (n=17, 15.9%), wound infections (n=13, 12.1%), and back pain (n=12, 11.2%). More detail on the reasons for ER visits is shown in Table 4. Of note, 25 of the 45 (56%) non-MMS patients had a visit reason involving pain, while 21 of the 62 (34%) MMS patients presented with pain as a concern. Note that a patient can have more than 1 reason for an ER visit.

**Table 3:** Ninety-day clinical outcomes.

90-day outcome	Non-MMS^a^ (n=336)			MMS (n=679)			Total (n=1015)			Univariate MMS:non-MMS			Multivariate MMS:non-MMS	
	n (%)	95% CI	n (%)		95% CI	n (%)		95% CI	OR^b^ (95% CI)		*P*	OR (95% CI)		*P*
ER^c^ visit	45 (13.4)	9.8-17	62 (9.1)		7-11.3	107 (10.5)		8.7-12.4	0.65 (0.43-0.98*)*		.04	0.68 (0.45-1.02)		.06
Readmission	30 (8.9)	5.9-12	55 (8.1)		6.1-10.2	85 (8.4)		6.7-10.1	0.90 (0.56-1.43)		.65	0.97 (0.6-1.55)		.88
Mild complication	32 (9.5)	6.4-12.7	56 (8.3)		6.2-10.3	88 (8.7)		6.9-10.4	0.82 (0.52-1.3)		.40	0.95 (0.59-1.51)		.82
Severe complication	43 (12.8)	9.2-16.4	66 (9.7)		7.5-12	109 (10.7)		8.8-12.6	0.72 (0.48-1.09)		.12	0.78 (0.52-1.19)		.25

^a^MMS: ManageMySurgery app.

^b^OR: odds ratio.

^c^ER: emergency room.

**Table 4:** Reasons for 90-day postoperative ER visits.

Reason for the ER^a^ visit	Non-MMS^b^ (n=45), n (%)	MMS (n=62), n (%)	Total (n=107), n (%)
Syncope/fall	5 (11.1)	12 (19.4)	17 (15.9)
Wound infection	4 (8.9)	9 (14.5)	13 (12.1)
Back pain	7 (15.6)	5 (8.1)	12 (11.2)
Limb pain	6 (13.3)	5 (8.1)	11 (10.2)
Chest pain	6 (13.3)	5 (8.1)	11 (10.2)
Neurological symptoms	3 (6.7)	4 (6.5)	7 (6.5)
Dyspnea	4 (8.9)	5 (8.1)	9 (8.4)
Leg swelling/DVT^c^	5 (11.1)	3 (4.8)	8 (7.5)
Abdominal pain	5 (11.1)	3 (4.8)	8 (7.5)
Dysphagia	0 (0.0)	4 (6.5)	4 (3.7)
Pain: unspecified	2 (4.4)	2 (3.2)	4 (3.7)
Urinary symptoms	1 (2.2)	3 (4.8)	4 (3.7)
Neck pain	2 (4.4)	2 (3.2)	4 (3.7)
Palpitations	0 (0.0)	2 (3.2)	2 (1.9)
Other	8 (17.8)	9 (14.5)	17 (15.9)
Unknown	0 (0.0)	2 (3.2)	2 (1.9)

^a^ER: emergency room.

^b^MMS: ManageMySurgery app.

^c^DVT: deep vein thrombosis.

## Discussion

### Principal Results

We present a study analyzing the postoperative clinical outcomes of patients who were invited to use a mobile digital health tool (MMS) before their elective spine surgery at a large, US academic medical center. In our univariate analysis, we found that compared to patients using traditional perioperative care, those using MMS had 35% lower odds of postoperative ER visits than did the non-MMS group and fewer severe postoperative complications in the 90 days after surgery. After adjustments for age and sex, there was a marginal improvement in ER visits in the MMS user group, with the MMS group having 32% lower odds of an ER visit than the non-MMS group, but this result did not show statistical significance (*P=*.06).

Our findings suggest that digital health solutions may assist in lowering adverse patient outcomes following spine surgery and may help reduce unnecessary health care resource utilization associated with ER visits. This is one of the first studies to document tendencies in acute outcomes associated with the use of a mobile health tool for patients undergoing spine surgery. Previously published research on mobile health solutions for spine surgery demonstrated the viability of using apps to engage patients in their recovery process and documented one such app that resulted in decreased pain scores when compared to traditional rehabilitation alone [30]. However, very few spine surgery apps have been linked with ER visits or complications. As a result, our findings support efforts to incorporate digital health tools into clinical practice, as they might help enhance objective outcomes through mass patient education.

### Comparisons With Prior Work

#### ER Visits

Postoperative pain has been blamed for the vast majority of unnecessary postoperative ER visits following spine surgery [4], which could cost billions of dollars per year. In orthopedic surgery, Kelly et al [31] discovered that the most prevalent reasons for ER visits following knee arthroplasty are pain and swelling. The authors then focused on pain and edema, changing discharge instructions to better educate patients and improve pain control, with the goal of lowering ER visits for these reasons [31]. Table 4 shows that of the 45 non-MMS patients who presented to the ER, 25 (56%) had a complaint of pain, compared to only 21 of the 62 (33.9%) MMS users with ER visits. Following spine surgery, postoperative discomfort in areas such as the neck, back, and limbs is a common and often benign occurrence. By establishing realistic expectations through patient education, digital health solutions such as MMS may remind patients of a normal postoperative course, potentially leading to fewer unnecessary postoperative pain visits. Such solutions may also encourage appropriate ER visits, as apps like MMS can educate patients about red flags that indicate a visit is necessary.

Additionally, patient engagement plays a role in reducing ER visits after various types of surgery. Close patient follow-up via scheduled phone calls or additional outpatient visits has been demonstrated to prevent avoidable ER utilization following surgery [32-34]. Improved patient education and awareness leads to lower emergency service utilization as patients' concerns are allayed by their health care providers. MMS keeps patients engaged throughout their surgical journeys by providing pre- and postoperative education, reminders, and surveys.

#### Readmissions and Postoperative Complications

There was no evidence of a significant reduction in readmissions among MMS users according to our analysis. Prior studies have found 90-day readmission rates of approximately 6%-10% following elective spine surgery [24,35]. Our study found that the MMS and non-MMS groups had readmission rates of 8.1% (55/679) and 8.9% (30/336), respectively.

Although our study did not reveal a statistically significant reduction in 90-day postoperative complications, it did find numerically fewer problems among MMS patients. Our complication rates are comparable to those reported in the literature for both MMS and non-MMS populations. Prior research on enhanced recovery after surgery pathways indicates that implementing these postoperative protocols has resulted in a reduction in the time of hospital stay following spine surgery but has not resulted in a reduction in complication rates [36,37]. Perhaps the numerically lower complication rates observed in our study cohort indicate that preoperative education provided by digital health, such as proper wound care and ambulation, may help patients prepare for surgery in such a way that they are less likely to experience preventable complications following surgery. Another possibility is that patients who used MMS were already healthier and more involved in their health, minimizing their susceptibility to problems; however, because we did not collect data on patient comorbidities, this will need to be investigated further in future studies.

#### Impact on Providers

Patient engagement strategies such as phone calls and more frequent follow-up visits place an increased burden on physicians and other health care practitioners, which is where mobile apps can help. MMS keeps patients involved in their surgical journeys by providing pre- and postoperative education, reminders, and surveys. Using a smartphone app can help relieve some of the strain on health care practitioners, allowing them to focus on more critical patient care responsibilities. For example, a practitioner having to administer the PROMIS-29 and Oswestry Disability Index at preoperative, 6-week, and 3-month timelines would have to administer 6 surveys per patient during the time period analyzed in this study. Instead, these can be completed automatically by patients without using limited provider time. Interestingly, it appears that while most patients did not view any FAQs, many logged into MMS 4 to 7 or more times, suggesting that they were spending time on the app completing surveys and other tasks when engaged with the platform. This suggests that MMS is helping to alleviate the time-intensive burden of survey completion from providers but that patients might prefer to receive information about their surgery directly from their providers.

A reduction in ER visits at large tertiary medical centers, such as the one where this study was conducted, may help alleviate ER overcrowding and enable more efficient workflows for emergency medicine providers, resulting in tangible benefits for both providers and other patients in need of emergency care. These benefits have been reported in other studies as well, with a systematic review of mobile health technologies for surgical patients reporting that mobile apps have been shown to reduce postoperative emergency visits, prevent inappropriate visits for wound checks, and improve adherence to postoperative rehabilitation [30]. In the realm of spine surgery, one study noted that many in-person follow-ups were avoided with the use of a mobile app, as it allowed issues such as pain control to be resolved remotely [38]. Our study thus expands on what other researchers have noted regarding the ability of digital health tools to reduce provider burden.

### Limitations

First, although our study found links between digital health use and fewer ER visits, our sample size was insufficient to establish statistical significance with a multivariable model. To have 80% power to detect a .05 level of significance with ER visit rates of 9.1% and 13.4% (from our results), a sample size of about 1700 patients would be required; this number would likely be higher for a multivariable study. Second, in our multivariable model, we did not account for patient comorbidities, which can affect outcomes. In a planned randomized controlled trial, we hope to address both of these limitations.

Third, rather than analyzing the level of engagement and its relationship to patient outcomes, we examined whether the patient used the app on a binary scale. This is a limitation of the app because we currently have no way of measuring the amount of time spent on it. Because it is impossible to predict when a patient will log out of MMS, a patient may open and use the app multiple times during a single login. We hope to address these issues in subsequent updates.

Finally, the findings are limited by the inherent biases of retrospective cohort studies. This demonstrates selection bias, as even nonusers of MMS were invited to download the app but never logged in or used it. Furthermore, the study is hampered by the inherent biases associated with electronic health record review, such as data entry errors, missing data from unconnected record systems, and discrepancies in chart review among reviewers. Future clinical research will be conducted to illustrate the impact of digital health technologies while addressing some of these possible constraints.

### Future Directions

This preliminary retrospective study provides data to suggest that the use of a digital health tool could help improve patient outcomes. In order to establish a more definitive link between MMS use and reductions in negative postoperative outcomes, we are planning a randomized controlled trial with MMS as the primary intervention for patients undergoing spine surgery. This trial would have a larger sample size, control for patient comorbidities, and use an intention-to-treat analysis to see if digital health tools can play a role in improving patient outcomes. Other future studies will involve analyzing subjective patient measures, such as the PROMIS-29 surveys given to patients within MMS.

### Conclusions

We have demonstrated the potential utility of a digital health platform (MMS) to improve health care utilization and patient outcomes in spine surgery, specifically demonstrating the tendency in reducing postoperative ER visits. Digital health platforms could prevent unnecessary ER visits by keeping patients engaged in their preparation for and recovery from major surgery as well as educating them on what a normal recovery looks like. This would relieve additional strain on patients, caregivers, providers, and the health care system as a whole. A randomized controlled trial is planned in the future to account for unmeasured confounders in this study.

## Appendix

Multimedia Appendix 1 Screenshot of the ManageMySurgery app with a description of the app's features.

Multimedia Appendix 2 Screenshot of the ManageMySurgery app patient and provider interfaces.

## Abbreviations

ACDF: Anterior Cervical Discectomy & Fusion
ER: emergency room
FAQs: frequently asked questions
HIPAA: Health Insurance Portability and Accountability Act
MMS: ManageMySurgery
PROMIS-29: Patient-Reported Outcomes Measurement Information System 29

## Data Availability

The data sets generated during and analyzed during this study are not publicly available in order to protect the patient health information found in the data but can be deidentified and shared by the corresponding author on reasonable request and with the approval of the institutional review board.
