# Supplementary figures and images for: Outcomes With a Mobile Digital Health Platform for Patients Undergoing Spine Surgery: Retrospective Analysis
Source: JMIR Perioper Med. 2022 Oct 26;5(1):e38690. doi: 10.2196/38690 (PMC9647464; doi:10.2196/38690)

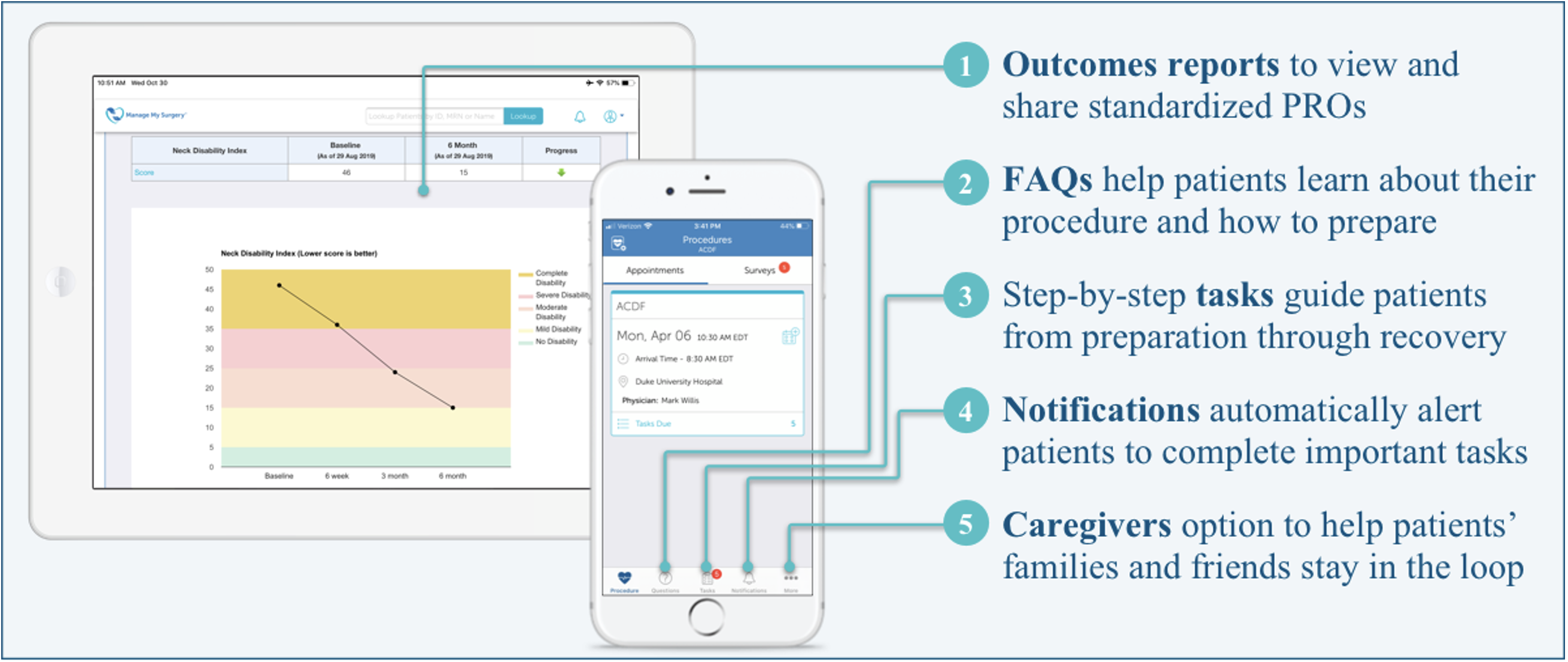

Supplement: Multimedia Appendix 1 [file periop_v5i1e38690_app1.png]

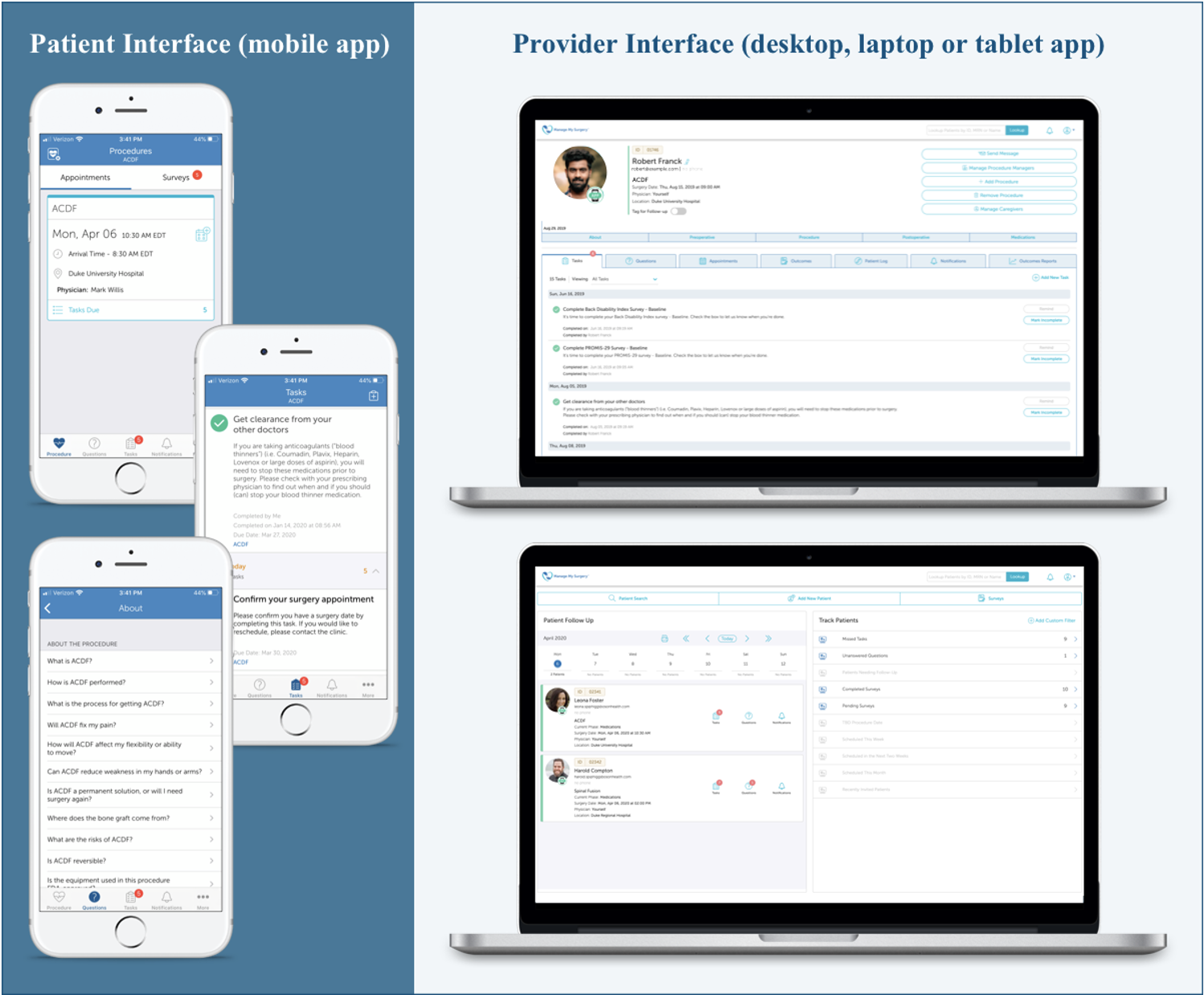

Supplement: Multimedia Appendix 2 [file periop_v5i1e38690_app2.png]
